# Flat Aesthetic Mastectomy Closure with the Angel Wing Technique to Address Lateral Adiposity: Technique and Outcome Analysis

**DOI:** 10.1155/2024/7349633

**Published:** 2024-03-25

**Authors:** E. Klenotic, D. Ochoa, K. Stephenson, C. Croswell, S. Sullivan, A. C. Sherman, R. Henry-Tillman

**Affiliations:** ^1^Division of Breast Surgical Oncology, Department of Surgery, University of Arkansas for Medical Sciences and the Winthrop P. Rockefeller Cancer Institute, Little Rock, AR, USA; ^2^Department of General Surgery, University of Arkansas for Medical Sciences, Little Rock, AR, USA; ^3^College of Medicine, University of Arkansas for Medical Sciences, Little Rock, AR, USA; ^4^Behavioral Medicine, University of Arkansas for Medical Sciences Winthrop P. Rockefeller Cancer Institute, Little Rock, AR, USA

## Abstract

**Introduction:**

Oncologic mastectomy in the setting of obesity poses challenges in achieving a flat closure that includes the lateral adiposity. The angel wing (AW) technique was developed to address this issue. We aim to demonstrate the safety of AW by evaluating incidence of arm lymphedema (AL) and decreased range of motion (dROM) in patients postmastectomy with and without the AW closure.

**Methods:**

We performed a single-center retrospective cohort study at an academic referral center of patients who underwent mastectomy with and without the AW technique from May 2014 to October 2022. Those who received breast reconstruction (immediate or delayed), partial mastectomy, and male patients were excluded. The presence of postoperative AL and dROM was evaluated. Subgroup analysis was performed for patient factors including BMI, extent of axillary surgery, PMRT, and pathologic stage.

**Results:**

A total of 390 patients met inclusion criteria. Of those, 173 (44.4%) underwent AW and 217 (55.6%) had non-AW mastectomy. Expectedly, the average BMI was significantly higher in the AW cohort (*p* < 0.0001). The overall rate of AL was 51/390 (13.1%), seen in 27 (15.6%) undergoing AW and 24 (11.1%) non-AW (*p* value = 0.18). While the rate of dROM within the cohort was 52/390 (13.1%), 27 (15.6%) underwent AW vs. 24 (11.1%) non-AW (*p* value = 0.22), resulting in no statistical significance between AW and non-AW mastectomy upon subsequent development of AL or dROM.

**Conclusion:**

Our study demonstrates the AW technique does not convey an increased risk of overall AL or dROM, even when considering known risk factors such as obesity, PMRT, and extent of axillary surgery. As we strive to provide our patients with improved surgical techniques for oncologic resection, we submit that this technique is a viable and safe option for achieving the goals of cosmesis with oncologic safety.

## 1. Introduction

As mastectomy rates have changed over the years, there is a growing interest in patients who desire mastectomy without reconstruction (“going flat”) [1]. However, oncologic mastectomy in the setting of obesity poses challenges in achieving an aesthetic flat closure (AFC) that include the lateral fat pad or fold, defined as the lateral adiposity. This lateral adiposity can cause chafing, pain, range of motion limitation, challenges in prosthetic fit, and dissatisfaction of appearance.

Several techniques to address lateral adiposity have been described. These techniques include the Y-shaped incision by Szynglarewicz et al. [2], the modified V-Y advancement technique by Gibbs et al. [3], the Fish-tail plasty by Hussien et al. [4], the modified M-plasty by Meybodi et al. [5], the double S technique by Steffens et al. [6], the tear-drop incision by Mirza et al. [7], and the L incision and lipoaspiration by El Hajj et al. [8]. The angel wing (AW) technique was first described by Hill and Henry-Tillman et al. in 2018 [9]. It is novel in that it avoids the frequently seen watershed area resulting in an increased risk of posterolateral junction skin ischemia or flap necrosis as well as providing an aesthetic linear scar. The previously described Y-shaped incision added two oblique incisions (two triangular-shaped flaps) to the traditional transverse Stewart mastectomy incision at the lateral part forming a Y-shape [2], and the modified V-Y advancement technique retracted the lateral apex of the mastectomy incision medially and secured the apex to the approximated transverse incision, followed by excision of the redundant superior and inferior tissues, forming a Y-shaped configuration upon closure [3]. The fish-tail plasty was similar. A stay suture is used to approximate the superior and inferior skin flap just lateral to the anterior axillary line, the lateral end of the mastectomy incision is brought medially to the existing stay suture, and the redundant skin is excised superiorly and inferiorly [4]. Within the modified M-plasty, the lipodermal flap of the M-plasty is de-epithelialized, pulled medially and secured to the pectoralis fascia according to the desired lateral contour flattening, also resulting in Y-shaped closure [5]. Of note, with these techniques employing a Y junction, skin necrosis is not infrequent. This occurs in 1.7–3.6% of patients, and they additionally exhibit an increased risk of dehiscence [2–5]. Other techniques avoid this Y junction, such as the double S technique [6], the tear-drop incision [7], and the L incision and lipoaspiration [8]. These all have their advantages and drawbacks. The double S technique employs two S-shaped incisions, superior and inferior to the nipple areolar complex. This enables the surgeon to shift the incision medially or laterally depending on the tumor location and the amount of lateral adiposity [6]. The tear-drop incision results in a broader incision laterally, including a segment of the axillary fat pad [7]. Of note, the double S technique and the tear-drop incision do not address the posterior aspect of the lateral adiposity. Conversely, the L incision and lipoaspiration include removal of a portion of the axillary fat pad and liposuction of the posterior axillary fat; however, the incision extends to the axillary crease resulting in an increased risk of lymphatic disruption [8].

**Figure 1:**
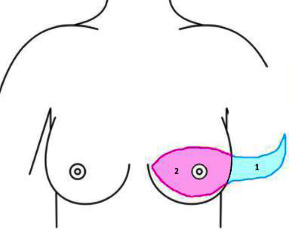
The angel wing technique. The AW technique consists of two parts, a lateral incision to address the redundant adipose tissue and the mastectomy incision.

**Figure 2:**
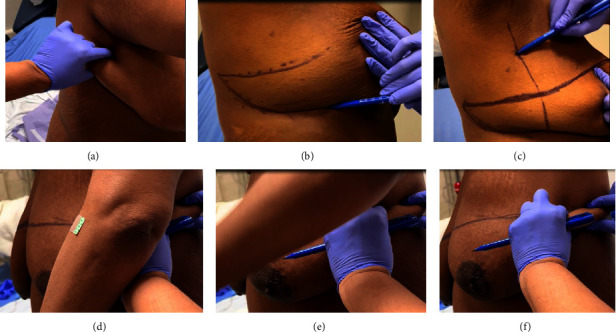
The angel wing marking. (a) Pinch test. (b) Two parallel curvilinear lines. (c) Anterior axillary line marking. (d–f) With the lateral adiposity “pinched,” the patient is asked to raise her arm to ensure that the lateral adiposity removed would not restrict full range of motion.

The AW technique pioneered by Henry-Tillman at the University of Arkansas Medical Sciences (UAMS) was developed to minimize the risk of posterolateral junctional skin ischemia or flap necrosis while addressing lateral adiposity, creating a linear scar that is aesthetically pleasing. The preliminary study revealed increased patient satisfaction and improved functional outcomes [9]. The AW technique consists of two parts, a lateral incision with superior and inferior flaps development to address the lateral adiposity and the mastectomy incision (see Figure 1). Preoperatively, the patient is evaluated in a standing or upright sitting position with her arm relaxed in front. A pinch test is done to assess the amount of lateral adiposity to be excised; with the lateral adiposity “pinched,” the patient is asked to raise her arm to avoid the range of motion dysfunction. The posterolateral end point is outlined based on the amount of lateral adiposity; therefore, the posterior end of the angel wing varies between patients. The AW portion of the procedure is composed of two parallel curvilinear lines with the superior line inferior to the axillary hairline and the inferior line extending from the posterior point to the sternal border along the entire inframammary crease. An anterior axillary line marking is drawn to ensure proper alignment during skin reapproximation. The mastectomy incision is then marked in the standard width to length ratio of 1 to 3, with the superior marking typically created at the level of the inframammary crease for a flat closure, yet without tension (see Figure 2).

**Figure 3:**
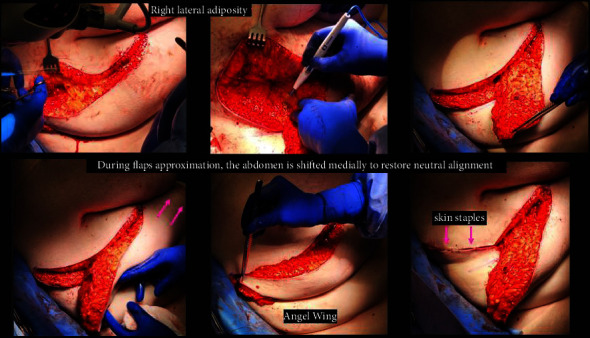
Intraoperative angel wing technique. The AW incision is created first, and thick subcutaneous flaps developed superiorly and inferiorly; the excess lateral adiposity is excised superficially to the latissimus dorsi fascia, keeping the fascia intact. During flap reapproximation, it is important to shift the ipsilateral abdominal wall medially to restore neutral body alignment. Skin staples are used to temporarily approximate the lateral skin for ease of flap alignment and to minimize blood loss.

**Figure 4:**
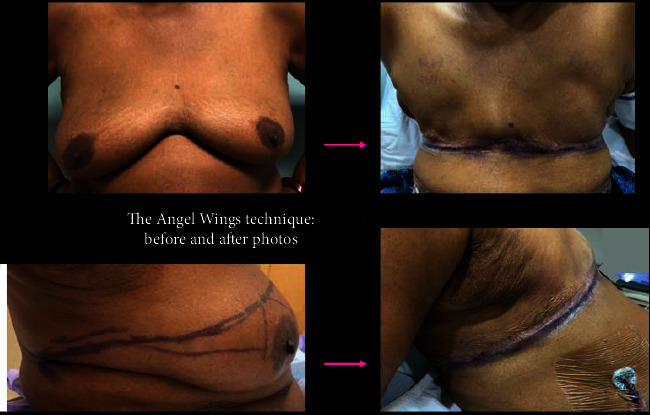
Mastectomy with angel wing technique: before and after. The lateral body contour is smooth and flat.

In the operating room, the patient is positioned in the supine position with arms extended 90 degrees, and sterile covers are used for the arms to allow medial arm retraction by an assistant. The AW incision is performed via a separate incision to the anatomical borders of the mastectomy. Thick subcutaneous flaps are developed superiorly and inferiorly, and the excess lateral adiposity is excised superficially to the latissimus dorsi fascia, keeping the fascia intact. Of note, during flap reapproximation, it is important to shift the ipsilateral abdominal wall medially to restore neutral body alignment. Skin staples are used to temporarily approximate the lateral skin for ease of flap alignment and to minimize blood loss. The mastectomy ± axillary portion of the procedure is then completed in a standard fashion, and a drain is placed in the mastectomy cavity. The incisions are closed with a single layer of 3−0 absorbable suture followed by skin adhesives (see Figure 3). Before and after photos are shown in Figure 4.

## 2. Methods

We performed a single-center retrospective cohort study at an academic tertiary referral center, evaluating all patients who underwent mastectomy with and without the AW technique, encompassing an 8.5-year period from May 1, 2014, to October 31, 2022. As this was a retrospective study employing deidentified data, the study was determined to be institutional review board (IRB) exempt as nonhuman subject research, and a waiver of consent was obtained by the University of Arkansas for Medical Sciences IRB. Eligible patients included all women who underwent mastectomy during the selected timeframe. Patients were identified through an EMR query of all who underwent a mastectomy through CPT codes 19303–19307 with and without tissue rearrangement codes 14301-14302. Patients with a history of breast reconstruction (immediate or delayed), partial mastectomy, and male patients were excluded. The presence of postoperative arm lymphedema (AL) and decreased range of motion (dROM) was determined by documentation in the center's postoperative breast surgery clinic. Descriptive statistics for the incidence of AL and dROM, as well as additional potentially associated factors (i.e., BMI, the extent of axillary surgery, axillary radiation, and pathologic stage), were performed, yielding medians with an interquartile range (IQR) for continuous variables and percentages for categorical ones. The association between the AW technique and the non-AW technique upon the development of AL and dROM was assessed through chi-square analysis. Analyses were conducted in SAS software, version 9.4 (SAS Institute, Cary, North Carolina, USA).

## 3. Results

A total of 390 patients met the inclusion criteria. Of those, 173 (44.4%) had AW performed and 217 (55.6%) underwent mastectomy without employing the AW technique. The average age was 61.1, and average BMI was 31.8 kg/m^2^. Eight patients underwent prophylactic mastectomy with the remainder performed for malignancy. Of note, ten had no axillary surgery. Of those who underwent axillary surgery (*n* = 380, 97.4%), 226 (57.9%) underwent sentinel node biopsy and 154 (39.5%) had axillary node dissection performed. In terms of pathologic staging, 295 (75.6%) were stage 0–II, 85 (21.8%) were III-IV, and 10 (2.6%) were not staged. Postmastectomy radiation therapy (PMRT) was performed in 138 (35.4%) patients. The AW and non-AW cohorts did not significantly differ except for BMI (*p* < 0.0001), with those undergoing AW exhibiting an average BMI of 35.3 kg/m^2^ and those without AW averaging 29 kg/m^2^ (see Table 1).

The overall AL rate was 13.1% (*n* = 51/390), seen in 15.6% (*n* = 27/173) undergoing the AW technique and 11.1% (*n* = 24/217) in those without the AW technique, and the development of AL did not significantly differ between the technique utilized (*p*=0.18). Of note, AL developed in 3.5% (*n* = 8/226) of those who underwent sentinel lymph node biopsy (SLNB) and 27.9% (*n* = 43/154) of axillary lymph node dissections (ALND). It occurred in 26.1% (*n* = 36/138) of those undergoing PMRT and 32.4% (*n* = 35/108) of those undergoing both ALND and PMRT. When subgroup analysis was performed, evaluating subpopulations within the cohort with varying BMI, extent of axillary surgery, presence of radiation therapy, and pathologic stage, there was no statistical difference in AL development within patient populations undergoing the AW technique compared to the non-AW technique, with the exception of an increased rate of AL in those undergoing the AW technique and subsequent postmastectomy radiation therapy (35% vs. 19.2%, *p*=0.04) (see Table 2).

The overall dROM rate was 13.3% (*n* = 52/390), seen in 11% (*n* = 19/173) undergoing AW vs. 15.2% (*n* = 33/217), and did not significantly differ between the technique utilized (*p*=0.22). When subgroup analysis was performed, there was no statistical difference in dROM development within patient populations undergoing the AW technique compared to the non-AW technique (see Table 3).

## 4. Discussion

**Figure 5:**
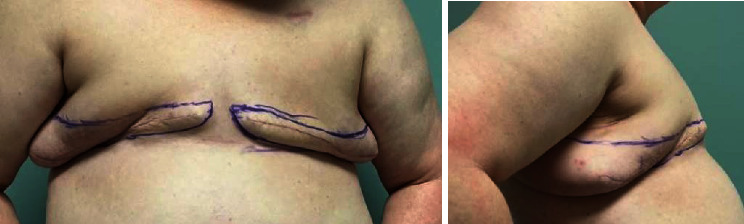
Lateral adiposity and redundant soft tissue postmastectomy. The patient presented after a simple mastectomy done at an outside facility. The arm adduction range of motion is limited due to the lateral adiposity.

Lateral adiposity in obese patients often conveys a challenging aesthetic flat closure in oncologic mastectomy. This lateral adiposity is often exaggerated and bothersome to patients postprocedure. Redundant soft tissue and lateral adiposity postmastectomy may cause decreased range of motion, chaffing, poor self-image, and difficulty with prosthesis (see Figure 5).

**Figure 6:**
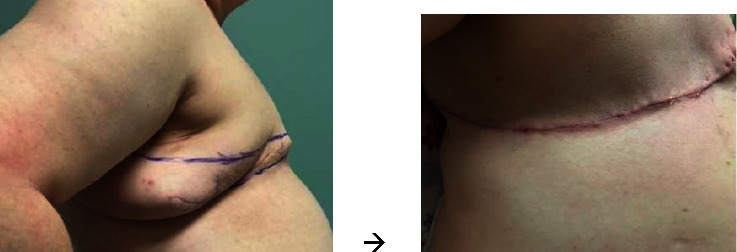
Scar revision with the angel wing technique. Angel wing is an excellent technique for mastectomy scar revision.

Many techniques have been developed to address this issue, including the M-plasty, Y-plasty, double S-plasty, advancement flaps, and staged liposuction. However, these procedures are often done by a plastic surgeon as a separate procedure after the mastectomy and are often associated with complications such as wound dehiscence and necrosis with a Y-shaped closure. The AW technique was developed at the University of Arkansas for Medical Sciences as another option to address lateral adiposity at the time of mastectomy. The angel wing technique is different from an extended dog ear revision in that it includes the entirety of lateral adiposity to the fold/crease, instead of just addressing the dog ear at the apex of the closure. The extension to incorporate the fat fold makes this technique a unique and novel approach in addressing the lateral adiposity. As it is performed at the time of mastectomy, it eliminates unnecessary additional visits and surgeries that may be seen as “cosmetic” procedures and may not be covered by insurance. This can have large repercussions upon patient satisfaction and quality of life, as many with lateral adiposity postmastectomy are reluctant to undergo additional procedures despite experiencing poor bra fitting, decreased range of motion, poor body image, and dissatisfaction after surgery. In addition, the AW technique is reproducible and simple, enabling those without formal plastic surgery training to easily adopt, with a learning curve of 4-5 cases. The AW is an excellent technique for mastectomy scar revision as well (see Figure 6).

As surgeons evaluate the potential of incorporating this technique into their practice, it is worth noting that the AW technique does increase the operative time depending on the amount of lateral adiposity. However, there are current procedural terminology codes (14301-14302) which may be used to compensate for the added time and effort of employing this technique. The AW technique does require a separate incision with flaps. While it does not prevent future breast reconstruction, if desired by patients, a latissimus dorsi flap may not be feasible, as some of the soft tissue over the latissimus has been removed. A previous study by Hill and Henry-Tillman et al. noted increased patient satisfaction and improved functional outcomes with use of the AW technique [9]. However, the rates of procedural complications such as lymphedema and decreased range of motion remained largely unknown.

Complications such as lymphedema, wound healing, and range of motion postmastectomy are known to be affected by age, BMI, stage, the extent of axillary surgery, chemotherapy, and postmastectomy radiation in addition to the procedural technique. Therefore, we set out to determine the incidence of these functional outcome complications within those undergoing mastectomies with and without the AW technique as related to these factors. Notably, this study confirmed the AW technique to be noninferior, without conveying significant increased risk of these complications. With regards to AL, the overall incidence was slightly higher in the AW cohort; however, there was no statistical significance between those undergoing the AW and non-AW techniques (*p*=0.18). When subgroup analysis was performed, evaluating subpopulations within the cohort with varying BMI, extent of axillary surgery, presence of radiation therapy, and pathologic stage, there was no statistical difference in AL development within patient populations undergoing the AW technique compared to the non-AW technique, with the exception of an increased rate of AL in those undergoing the AW technique and subsequent postmastectomy radiation therapy (35% vs. 19.2%, *p*=0.04). This could be explained by several factors including low number of patients, the possibility of extended radiation field to include the AW incision, and the compounding risk factor of obesity, as those within the AW cohort unsurprisingly exhibited a higher BMI (*p* > 0.0001). It is important to discuss with the radiation oncology team to prevent extensive radiation at the AW incision beyond the anatomic borders of the breast and axilla. Obesity is a known risk factor for AL development [10, 11], and others have proposed the relationship between obesity and lymphedema is reciprocal, as obesity impairs lymphatic transport and impaired lymphatic function promotes adipose deposition [10]. In addition, in our experience, the extension of the incision into the angel wing does not lead to any additional neurosensory changes or sequelae. The extension incorporates only subcutaneous adipose tissue and overlying skin. It does not extend to the depth of nerves in this portion of the dissection, and there have been no additional neurosensory impacts related to this portion of the surgery.

As the AW technique involves removal of lateral adiposity and skin inferior to the axilla, there lies an inherent risk of dROM. Of note, the technique includes performance of a “pinch test” in preoperative marking, with the arm elevated to ensure an aesthetic flat closure without conveying risk for tension and dROM postoperatively. Notably, dROM was seen less frequently within the AW cohort, seen in 11% (*n* = 19/173) undergoing AW vs. 15.2% (*n* = 33/217); however, this difference was also not significant (*p*=0.22). When subgroup analysis was performed, there was no statistical difference in dROM development within patient populations undergoing the AW technique compared to the non-AW technique.

Of note, the current study is restricted by its single-center, retrospective design. Therefore, these results may not extend to differing populations and providers less familiar with the technique. This technique has been taught to over 20 breast surgical oncology fellows trained at UAMS, and future studies may include their outcomes. In addition, as a large portion of our patients complete their adjuvant treatments elsewhere, data regarding adjuvant treatments such as chemotherapy and the extent of radiation treatment were limited and thus not included in this study. In addition, incorporation of additional techniques such as axillary reverse mapping, which has been reported to reduce risk of lymphedema, can also be evaluated in future studies.

## 5. Conclusion

In conclusion, the AW technique is a safe and effective method for addressing lateral adiposity and achieving an aesthetic flat closure, without conveying an increased risk of lymphedema or the effect on the range of motion. As we strive to provide our patients with improved surgical techniques for oncologic resection, we submit that this technique is a viable and safe option for achieving the goal of cosmesis with oncologic safety without incurring increased risk of functional complication.

## Figures and Tables

**Table 1:** Demographics of angel wing versus nonangel wing technique cohort.

Variables	Demographics			
	Cohort	AW	Non-AW	
	*N* = 390	*n* = 173 (44.4%)	*n* = 217 (55.6%)	
BMI				
<25	76 (19.5%)	16 (9.3%)	60 (27.6%)	*p* < 0.0001
25–29.9	104 (26.7%)	33 (19.2%)	71 (32.7%)	
>30	210 (53.8%)	124 (72.1%)	86 (39.6%)	
Axillary surgery				
None	10 (2.6%)	6 (3.5%)	4 (1.8%)	*p*=0.13
SLNB	226 (57.9%)	91 (52.9%)	135 (62.2%)	
ALND	154 (39.5%)	76 (44.2%)	78 (35.9%)	
# of nodes removed				
<10	294 (75.4%)	130 (75.6%)	164 (75.6%)	*p*=0.87
10–19	76 (19.5%)	33 (19.2%)	43 (19.8%)	
>20 nodes	20 (5.1%)	10 (5.8%)	10 (4.6%)	
PMRT				
Yes	138 (35.4%)	60 (34.9%)	78 (35.9%)	*p*=0.80
No	252 (64.6%)	113 (65.7%)	139 (64.1%)	
PMRT + ALND	108 (27.7%)	50 (28.9%)	58 (26.7%)	NA
Stage				
0	52 (13.3%)	24 (14.0%)	28 (12.9%)	*p*=0.26
I	136 (34.9%)	50 (29.1%)	86 (39.6%)	
II	107 (27.4%)	53 (30.8%)	54 (24.9%)	
III	64 (16.4%)	32 (18.6%)	32 (14.7%)	
IV	21 (5.4%)	10 (5.8%)	11 (5.1%)	
N/A	10 (2.6%)	4 (2.3%)	6 (2.8%)	

Demographic information of those who underwent the angel wing and nonangel wing technique.

**Table 2:** Subgroup analysis of angel wing vs nonangel wing technique and subsequent arm lymphedema development.

Variables	Lymphedema			
	Cohort	AW	Non-AW	*p*=0.18
	*n* = 51	*n* = 27	24	
	(13.1%)	(15.6%)	(11.1%)	
BMI				
<25	6 (7.9%)	3 (18.8%)	3 (5%)	*p*=0.07
25–29.9	16 (15.4%)	5 (15.2%)	11 (15.5%)	*p*=0.96
>30	29 (13.8%)	19 (15.3%)	10 (11.6%)	*p*=0.45
Axillary surgery				
None	0 (0%)	0 (0%)	0 (0%)	NA
SLNB	8 (3.5%)	4 (4.4%)	4 (3.0%)	*p*=0.57
ALND	43 (27.9%)	23 (30.3%)	20 (25.6%)	*p*=0.52
# of nodes removed				
<10	22 (7.5%)	12 (9.2%)	10 (6.1%)	*p*=0.31
10–19	25 (32.9%)	13 (39.4%)	12 (27.9%)	*p*=0.29
>20 nodes	4 (20%)	2 (20%)	2 (20%)	*p*=1.0
PMRT				
Yes	36 (26.1%)	21 (35%)	15 (19.2%)	*p*=0.04
No	15 (6.0%)	6 (5.3%)	9 (6.5%)	*p*=0.7
PMRT + ALND	35 (32.4%)	20 (40%)	15 (25.9%)	*p*=0.12
Stage				
0	4 (7.7%)	3 (12.5%)	1 (3.6%)	*p*=0.23
I	10 (7.4%)	2 (4%)	8 (9.3%)	*p*=0.25
II	14 (13.1%)	10 (18.9%)	4 (7.4%)	*p*=0.08
III	15 (23.4%)	8 (24%)	7 (21.9%)	*p*=0.77
IV	8 (38.1%)	4 (40%)	4 (36.4%)	*p*=0.86
N/A	0 (0%)	0 (0%)	0 (0%)	NA

**Table 3:** Subgroup analysis of the angel wing vs nonangel wing technique and decreased range of motion.

Variables	Decreased range of motion			
	Cohort	AW	Non-AW	*p*=0.22
	*n* = 52 (68.4%)	*n* = 19 (11%)	*n* = 33 (15.2%)	
BMI				
<25	7 (9.2%)	(0%)	7 (11.7%)	NA
25–29.9	18 (17.3%)	6 (18.2%)	12 (16.9%)	*p*=0.87
>30	27 (12.9%)	13 (10.5%)	14 (16.3%)	*p*=0.22
Axillary surgery				
None	1 (10%)	1 (16.7%)	0 (0%)	NA
SLNB	14 (6.2%)	4 (4.4%)	10 (7.4%)	*p*=0.36
ALND	37 (24.0%)	14 (18.4%)	23 (29.5%)	*p*=0.11
# of nodes removed				
<10	25 (8.5%)	11 (8.5%)	14 (8.5%)	*p*=0.98
10–19	24 (31.6%)	7 (21.2%)	17 (39.5%)	*p*=0.09
>20 nodes	3 (15%)	1 (10%)	2 (20%)	*p*=0.94
PMRT				
Yes	32 (23.2%)	11 (18.3%)	21 (26.9%)	*p*=0.24
No	20 (7.9%)	8 (7.1%)	12 (8.6%)	*p*=0.65
PMRT + ALND	29 (26.9%)	10 (20%)	19 (32.8%)	*p*=0.14
Stage				
0	3 (5.8%)	(0%)	3 (10.7%)	NA
I	10 (7.4%)	5 (10%)	5 (5.8%)	*p*=0.37
II	15 (15.0%)	4 (7.5%)	12 (22.2%)	*p*=0.03
III	17 (26.6%)	8 (25%)	9 (28.1%)	*p*=0.08
IV	5 (23.8%)	1 (10%)	4 (36.4%)	*p*=16
N/A	1 (10%)	1 (25%)	0 (0%)	NA

## Data Availability

Data in this study are available upon request. Data were obtained from the University of Arkansas for Medical Sciences Electronic Medical Record and stored in a password protected Excel spreadsheet with patient identifiers deleted in an encrypted partition of an institutional computer, accessible only to the study's investigators.
